# The end-use quality of wheat can be enhanced by optimal water management without incurring yield loss

**DOI:** 10.3389/fpls.2022.1030763

**Published:** 2022-11-10

**Authors:** Kun Sheng, Lina Xu, Mingxia Wang, Heng Lei, Aiwang Duan

**Affiliations:** ^1^ School of Hydraulic Engineering, Yellow River Conservancy Technical Institute, Kaifeng, China; ^2^ College of Life Science and Technology, Henan Institute of Science and Technology, Xinxiang, China; ^3^ Farmland Irrigation Research Institute, Chinese Academy of Agricultural Sciences, Xinxiang, China

**Keywords:** wheat quality, water deficit, regulated deficit irrigation, grain growth, grain nitrogen content

## Abstract

In China, water-saving irrigation is playing important roles in ensuring food security, and improving wheat quality. A barrel experiment was conducted with three winter wheat (Triticum aestivum L.) genotypes and two irrigation pattens to examine the effects of regulated deficit irrigation (RDI) on wheat grain yield, water-use efficiency (WUE), and grain quality. In order to accurately control the soil water content, wheat was planted in the iron barrels set under a rainproof shelter, and the soil water content in the iron barrel was controlled by gravity method. The mechanisms whereby water management influences the end-use functional properties of wheat grain were also investigated. The results revealed that RDI improved the end-use functional properties of wheat and WUE, without significant yield loss (less than 3%). Moderate water deficit (60% to 65% field capacity) before jointing and during the late grain-filling stage combined with a slight water deficit (65% to 70% field capacity) from jointing to booting increased grain quality and WUE. The observed non-significant reduction in wheat yield associated with RDI may be attributed to higher rate of photosynthesis during the early stage of grain development and higher rate of transfer of carbohydrates from vegetative organs to grains during the later stage. By triggering an earlier rapid transfer of nitrogen deposited in vegetative organs, RDI enhances grain nitrogen content, which in turn could enhance dough elasticity, given the positive correlation between grain nitrogen content and dough midline peak value. Our results also indicate that the effects of RDI on grain quality are genotype dependent. Therefore, the grain end-use quality of some specific wheat genotypes may be enhanced without incurring yield loss by an optimal water management.

## 1 Introduction

It will be necessary for increasing continually food supplies to meet the demands of the growing world population in the future, and ensuring food security has accordingly become a primary focus of human society. In turn, food security is dependent on dependable supply of water resources (Kang et al., 2017), which is negatively affected by changes in agricultural water supply associated with the changing climate. It has been estimated that food production should increase at least 30% in order to meet the nutritional demands of the growing population in China. However, the average water resource amount per capita in China is less than one-third of the world average value, which shows that ensuring agricultural water supply in China is extremely important for food security.

In China, wheat is one of the most important cultivated food crops, with an annual production of more than 134 million tons (FAO, 2020), ranking third after corn and rice in terms of productivity. Wheat is primarily planted in northern China, in which only 19% of the country’s total water resources is distributed. Moreover, duo to the main precipitation period generally does not coincide with the peak water demand stages in wheat production, the wheat yield and quality are often affected significantly by drought in the main production regions (Zaveri and Lobell, 2019). Irrigation plays prominent roles in ensuring stable yield and high grain quality in wheat production in China. Therefore, to ensure synchronously water and food security in China, it is essential to understand the response mechanisms of wheat yield and quality to farmland water management patterns.

Regulated deficit irrigation (RDI), a water-saving irrigation method widely adopted in China, can be employed to reduce water consumption without causing a significant wheat yield lose. Under RDI, a limited water deficit is imposed during a given specific growth stage to control the redundant growth of vegetative organs, thereby promoting the distribution of photosynthates to reproductive organs, which is benefit for increasing economic yield and conserving water consumption. The successful application of RDI is dependent closely on understanding of the effects of soil moisture management on wheat yield and quality, as well as the associated physiological mechanisms. Meng et al. (2016) have previously suggested that a slight or moderate water deficit prior to the stem elongation stage, or a slight water deficit at the grain-filling stage, promotes high yield and water conservation in winter wheat. Yan et al. (2019) found that a mild water deficit could enhance grain filling rate and productivity of winter wheat. Furthermore, it has previously been established that to obtain high wheat grain yields, priority should be placed on ensuring irrigation during the jointing and filling periods (Mu et al., 2021a). A slight or moderate water deficit has been demonstrated to increase yield by inhibiting vegetative growth, increasing the harvest index (HI), and promoting growth compensation after the water stress was relieved in the next growth stage.

Numerous studies have been conducted to examine the effects of optimal water management (i.e., RDI) during entire growth season on grain protein content in wheat, and it has been established that generally, there is a negative linear correlation between grain protein content and grain yield. However, few studies have sought to evaluate the effects of RDI on the end-use functional properties of wheat.

The end-use functional properties of wheat, which determine the type and quality of the associated food products, are mainly determined by the content and characteristics of grain protein and starch in wheat grain. For example, protein characteristics, including content, composition, and polymerization, play important roles in determining bread and hard-bite noodle quality (Hou et al., 2013), whereas the swelling and pasting properties of starch are important determinants of soft noodle quality (e.g., udon). Furthermore, it has been demonstrated that protein content is positively correlated with bread volume (Dowell et al., 2008; Hu et al., 2021), but negatively correlated with noodle surface smoothness and visual brightness are protein content (Ohm et al., 2008). In addition, the proportion of low molecular weight glutenins/gliadins has shown a positively correlated with the hardness, gumminess, and chewiness of cooked Chinese hard-bite white-salted noodles (Hou et al., 2013), whereas wheat starches that confer high peak viscosity, high swelling potential, low pasting temperature, and high breakdown have been established to be associated closely with the superior quality of Japanese white-salted noodles (Noda et al., 2001).

Drought stress has significant effects on the parameters of Mixograph and GI. For example, the mixing characteristics associated with dough strength (e.g., midline peak value) have been shown to be increased, whereas the midline right slope is not significantly influenced by drought stress at the soft dough stage (Labuschagne and Moloi, 2015). Furthermore, irrigation regime plays an important role in determining the end-use quality of a given wheat genotype, and GI appears to be predominantly reduced under an abundant moisture regime (Gil et al., 2011). Flagella et al. (2010) demonstrated that terminal water stress during grain filling consistently enhances gluten strength, as indicated by GI. Li et al. (2013) detected a significant increase in gluten strength, although no significant increase in the midline peak time. However, there are few studies for examining the influence of RDI on mixograph parameters and the GI, and little knowledge on the mechanisms whereby water management modifies the end-use functional properties of wheat.

Grain protein concentration (GPC), proportional composition of protein, and protein polymerization are influenced to varying degrees by the characteristics of grain dry matter and nitrogen (N) accumulation. GPC, expressed as the ratio of protein weight to dry matter weight, is determined by the characteristics of both grain filling and grain N accumulation. In this regard, given that grain protein content is diluted by non-nitrogenous compounds as grain yield increases, yield is negatively correlated with protein concentration (Moral et al., 2007; Graziano et al., 2019; Tatar et al., 2020; Santis et al., 2021). However, the relationship between grain yield and GPC changes in response to different water treatments (Meng et al., 2016). Given the allometric scaling relationships between protein fraction composition and total quantity of N per grain (Plessis et al., 2013; Ferrise et al., 2015), the proportional composition of protein is primarily dependent on the total quantity of N per grain (Triboï et al., 2003). As total protein content increased, the ratio of low molecular weight to high molecular weight glutenins decreased consistently under water deficit occurred throughout the growing season (Flagella et al., 2010; Giuliani et al., 2015). Moreover, given that the glutenin polymer backbone is composed of high molecular weight glutenins, the content of these glutenins affects gluten strength. Accordingly, understanding the influences of water deficit at different wheat growing stages on the characteristics of grain dry matter and N accumulation are essential for gaining an insight on the physiological mechanisms whereby RDI affects GPC, composition, and protein polymerization.

Grain dry matter and N accumulation are also influenced by sucrose and N concentration in plants. The characteristics of grain dry matter accumulation is predominantly associated with starch, which accounts for more than 80% of the total grain dry matter (Evers et al., 1999). During grain filling in wheat, photoassimilates in leaves and carbohydrates in vegetative organs are transported to grains in the form of sucrose, providing a source of carbon for starch accumulation in the grains (Dong and Beckles, 2019; Zhang et al., 2021), with the translocation of assimilate from source to sink influencing the accumulation of grain dry matter (Ma et al., 1996). In this context, it has previously been established that a mild desiccation of soil enhances whole-plant senescence, thereby promoting a more rapid and efficient remobilization of carbon from vegetative tissues to grains, and accelerating the rate of grain-filling (Yang and Zhang, 2006). Moreover, the source of N incorporated into filling grains is assumed to be derived from the N remobilized from leaves (Kong et al., 2016). The accumulation of grain N occurs almost simultaneously with the decrease of leaf N content. It has also been reported that water deficit enhances N remobilization efficiency in wheat (Li et al., 2021). Consequently, a better understanding of the effects of water deficit on the changes in sucrose and N concentrations in different organs is important for clarifying the physiological mechanism whereby RDI influences grain filling and N accumulation characteristics.

In this study, we investigated the effects of RDI on wheat grain yield, water-use efficiency (WUE), and the grain quality traits e.g. GPC, GI, midline peak time (MPT), midline peak value (MPV), and right of midline peak slope (MRS), to demonstrate that optimal water management can enhance grain quality and WUE in the absence of any apparent reduction in grain yield. In addition, we also examined the mechanisms whereby water management influences the end-use functional properties of wheat. The main hypotheses in present study are as follows: (a) grain quality and WUE may be improved by optimal water management without apparent yield loss, (b) moderate water stress may increase deposit of carbohydrates and nitrogen in grain, and (c) grain nitrogen content may enhance grain end-use quality.

## 2 Material and methods

### 2.1 Experimental site

The experiment was carried out during the 2017–2018 growing season at the experimental station of the Farmland Irrigation Research Institute, Chinese Academy of Agricultural Sciences (35°19′N, 113°53′E), located in Xinxiang City, Henan Province, P. R. China. In order to accurately control soil water content (SWC), wheat was planted in the iron barrels under the rainproof shelter. The SWC in the iron barrel was controlled by gravity method, and irrigation was applied appropriately to regulate the SWC went to upper limit when the SWC was below lower limit.

**Figure 1:**
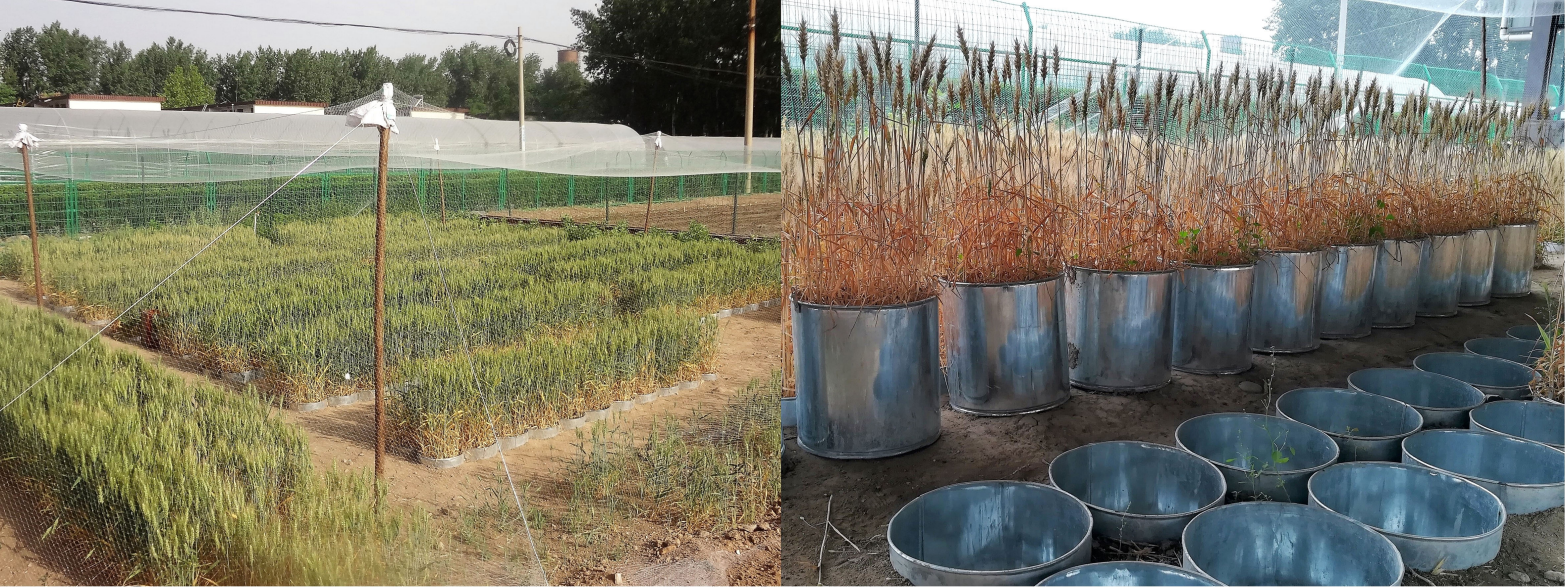
Photos of the experiment at ripening.

The iron barrel comprised an inner and outer layer, and the inner layer was closely nested in the outer layer (**Figure 1**). During the experiment, the outer barrel was buried 25 cm below ground level to maintain the temperature of soil within the barrel close to that of the surrounding soil. The inner barrel (30 cm diameter × 30 cm depth), which could be readily weighed, contained 20.5 kg (dry weight) of sandy loam soil. The SWC in the inner barrel was obtained by determining the gross weight of the inner barrel and plant fresh weight. SWC in the inner barrel = water weight in the inner barrel/dry soil weight in the inner barrel; water weight in the inner barrel = (inner barrel weight + wet soil weight in the inner barrel + plant fresh weight) – (inner barrel weight + dry soil weight in the inner barrel); inner barrel weight was 1.5 kg. Prior to water treatment, assuming that the fresh weight of plants in each water treatment was the same, we determined the fresh weight values of plants by measuring 10 plants from each variety. Following the water treatment, five plants were sampled for each water treatment and weighed at each growth stage. SWC was measured at weekly intervals prior to the jointing stage, at 3-day intervals from jointing to anthesis, and daily during the grain-filling stage.

### 2.2 Experimental site

**Figure 2:**
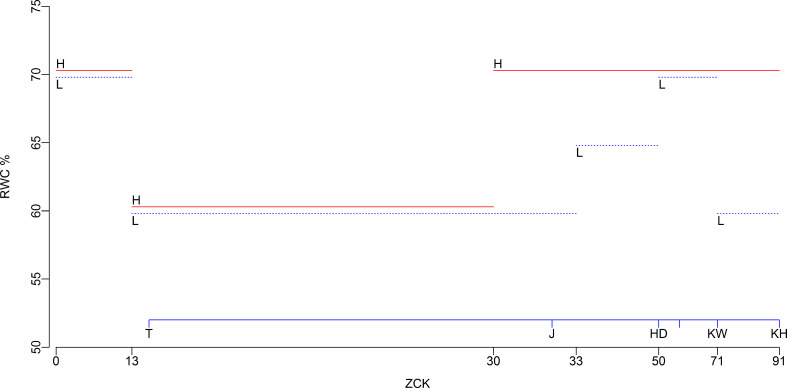
The lower limit of soil water content for water treatments. Red and blue lines indicate high-and low-water treatments, respectively. RWC, relative soil water content, percentage of field water capacity; H, high-water treatments; L, low-water treatments; ZCK, Zadoks-Chang-Konzak wheat developmental scale; T, tillering; J, jointing; HD, heading; KW, watery-ripe kernel; KH, hard kernel.

Two water treatments were utilized in this experiment, and the lower limit of SWC for water treatment is shown in **Figure 2** (Meng et al., 2016; Mu et al., 2021a). For high-water treatments (H, red lines), SWCs were no less than 70% of the field capacity (FC) from the sowing period to the seedling period (Zadoks-Chang-Konzak wheat developmental scale, ZCK 13), 60% to 65% FC from tillering (ZCK 21) to the erect leaf sheath stage (ZCK 30), and 70% to 75% FC from jointing (ZCK 31) to the hard kernel stage (ZCK 92). For low-water treatments (L, blue lines), SWCs were no less than 70% FC from sowing to the seedling period (ZCK 13), 60% to 65% FC from tillering (ZCK 21) to the visible third node stage (ZCK 33), 65% to 70% FC from the visible fourth node (ZCK 34) to booting (ZCK 49) stages, 70% to 75% FC from the heading (ZCK 50) to watery-ripe kernel (ZCK 72) stages, and 60% to 65% FC from the early milky-ripe (ZCK 73) to hard kernel (ZCK 92) stages.

For the purposes of the present study, we used three wheat genotypes, Xinmai-26 (XM26), Xinmai-49 (XM49), and Zhoumai-18 (ZM18), whose MPTs were 12, 8, and 4 min, respectively. These genotypes can thus be considered to be representative of very strong gluten, strong, and medium-strong cultivars, respectively. Among these, XM26 whose high molecular weight glutenin subunits were 1, 7 + 8, and 5 + 10, and ZM18 whose yield stability outperform other cultivars are widely cultivated in the Huang-Huai-Hai Plain of China. Whereas, XM49 is a new breeding line with a higher thousand-grain weights (TGW).

The treatments were set up using a split-plot design, with three replicates. The barrels were grouped into three blocks, each distributed in two plots, with each plot being subdivided into three subplots, each containing three barrels. Water treatments were randomly assigned to the plots within each block and genotypes were randomly assigned to the subplots within each plot.

### 2.3 Experimental procedure

Fertilizers (1.7 g N, 1.13 g P_2_O_5_, and 0.56 g K_2_O) were applied to each barrel. Half of the N was applied prior to sowing, with the remainder being applied at the jointing stage. Wheat seeds were sown on October 18, 2017, and plants were harvested on May 21, 2018. In each barrel, we sowed 36 seeds at a depth of 3 cm, which were thinned to 12 seedlings per barrel at the three-leaf stage.

At 3- to 5-day intervals post anthesis, three spikes bearing flag leaves were cut from the plants, from which the grains were harvested by hand and dried with the leaves at 80°C for 24 h. The mean dry weight of a single kernel was determined after weighing all kernels (approximately 100 kernels). Following these weight determinations, the dried grains, flag leaves, and rachises were ground to determine the sucrose and nitrogen contents.

In each sub-plot, one barrel of plants without destructive sampling was reserved for harvesting, with all plants in each of the barrels being harvested for grain yield determination. After weighing, the grains were ground for quality determination. For each barrel, we counted the number of spikes, and obtained measurements for total biomass (BM), HI, number of grains per spike (NGS), number of spikelets per spike (NSS), and number of infertile spikelets per spike (NIS) from 10 plants. TGWs were estimated from the measurements of three 200-grain samples.

Wheat evapotranspiration (ET) was determined using a soil water balance equation:


Eq. [1] ET=I+P+ΔS+ΔWS+ΔWV

Where I is the total irrigation amount (mm), P precipitation (mm), ΔS change of soil water storage (mm), ΔWS water exchange with outside through surface ways, and ΔWV water exchange with outside through vertical ways in a barrel during the whole wheat growing season. For the given situation in this experiment, the P, ΔWS, ΔWV may be considered as zero, and then the Eq. [1] may be simplified as follows:


Eq. [2] ET=I+ΔS

The WUE was calculated with following formula:


Eq. [3] WUE=GY/ET

Where GY is the grain yield.

GPC was determined using the micro-Kjeldahl method according to the AACC Method 46-11A. GN (mg/grain) = GPC × grain weight, and sucrose content was measured using resorcinol. GI values were determined based on the ICC standard No. 158 using a Perten Glutomatic 2200 Instrument (Perten, Sweden). Flour mixing characteristics were evaluated using a 10 g Mixograph (National Mfg. Div., TMCO, Lincoln, NE, USA) with an absorption of 0.625 mL/g (Peterson and Graybosch, 1992), according to the approved AACC Method 54-40A, 2002. MPT, MPV, and MRS were analyzed based on the correlations between the mixograph and baking parameters (Wikstrom and Bohlin, 1996; Chung et al., 2001; Neacşu et al., 2009).

### 2.4 Statistical analyses

Data analysis and plotting were performed by using R 4.1.3 software (R Foundation for Statistical Computing, Vienna, Austria) and RStudio 2022.02.1 + 461 integrated development environment. Data were analyzed using an analysis of variance (ANOVA), with means being compared using a least significant difference (LSD) test at the 5% level of significance. Pearson correlation coefficients among yield, ET, and quality traits were calculated using the software package “psych”, correlation diagnosis was performed using the package “corrgram”, and dynamic curves of sucrose and nitrogen concentration were plotted using the package “ggplot2” with the “loess” method.

The grain weight data were fitted using a re-parameterized Richards growth model, with the statistical expectation of the Richards model being expressed using Eq. [4] (Richards, 1959).


Eq. [4] $$\text{Q(t})\;=\text{K }\cdot \text{ (}1-\text{a}\cdot {\text{e}}^{-\text{bt}}{)}^{\frac{1}{1\;–\text{ c}}}$$

Where Q is the quantity of grain weight; t is the number of days after anthesis (DAA); K is the final value of Q approached as t→*∞*  ; and a, b, and c are coefficients determined by regression. The parameter c controls the inflection values. The maximum rate of accumulation (R) was defined as the derivative of the point of inflection, and the duration of grain filling (t95) was defined as the duration, from anthesis, in which 95% of K is accumulated (Ferrise et al., 2015).


Eq. [5] $$\text{R }=\text{K}\cdot \text{b}\cdot {\text{c}}^{\frac{\text{c}}{1-\text{c}}}$$

Eq. [6] $$\text{t}95\;=\;-\frac{1}{\text{b}}\ln\left[\frac{1-0.95^{\left(1-\text{c}\right)}}{\text{a}}\right]$$

Substituting Eq. [5]and Eq. [6] into Eq. [4] yields the re-parameterized Richards growth model shown in Eq. [7]:


Eq. [7] $$\text{Q(t})\;=\text{K }\cdot \;{\left\{1+[0.95^{\left(1-\text{c}\right)}-1]\cdot {\text{e}}^{\left[\frac{\text{R}\cdot (\text{t}95-\text{t})}{\text{K}\cdot {\text{c}}^{\frac{\text{c}}{1-\text{c}}}}\right]}\right\}}^{\frac{1}{1\;-\text{ c}}}$$

When c = 2, Eq. [7] becomes a three-parameter logistic model (Triboï et al., 2003). The nonlinear effect models for grain weight were fitted using the “nlst” function of the R/nlme package (Pinheiro and Bates, 2000). Within-barrel heteroscedasticity was modeled using a power-variance function, and alternative models were evaluated using the likelihood ratio test, log-likelihood, Akaike information criterion (AIC), Bayesian information criterion (BIC), and residual variance (Razali et al., 2015). Smaller AIC, BIC, and residual variance values indicate a better fit of the model to the experimental data (Aggrey, 2009). Finally, the parameter c in Eq. [7] was treated as a purely fixed effect and assigned a value of 2.4.

## 3 Results

### 3.1 Grain yield, water-use efficiency, and harvest index of winter wheat

#### 3.1.1 Grain yield and yield components

The grain yield and yield components of wheat exposed to the different irrigation treatments are shown in **Table 1**. The genotype (G) and genotype-by-water interaction (G×W) were found to have significant effects on grain yield, whereas the effects of water (W) on grain yield did not reach the significant level (P > 0.05). Relative to the value obtained in response to the H, the reduction in grain yield under L was 2.96% (1.4g). Compared with H treatment, there were significant reductions of 7.57% and 7.47% in the number of spikes per barrel (NSB) and NGS, respectively, in the L treatment. Although NIS was not significantly affected by the irrigation regime, a 6.43% reduction of NSS was recorded under L treatment, compared with that under H treatment. In addition, the TGW was found to be significantly influenced by water treatment levels, with an increase of 3.14% under L treatment.

**Table 1:** Grain yield, yield components, water-use efficiency and harvest index of winter wheat genotypes under the different levels of irrigation.

	GY	ET	WUE	BM	HI	NSB	TGW	NGS	NSS	NIS
Water(W)	ns	**	**	**	**	**	**	*	**	ns
H	47.2	35.92a	1.3183b	113.14a	41.64b	42.67a	45.19b	30.489a	18.133a	3.322
L	45.8	31.33b	1.472a	104.11b	44.0a	39.44b	46.61a	28.211b	16.967b	3.167
Genotype(G)	**	ns	**	*	ns	ns	**	*	ns	ns
XM26	49.84a	33.59	1.492a	113.55a	44	42.33	42.46c	30a	17.317	2.85
XM49	39.69b	34.86	1.1443b	94.5b	42.02	40.67	49.88a	27.983b	17.367	3.8
ZM18	49.97a	32.42	1.5491a	117.83a	42.44	40.17	45.38b	30.067a	17.967	3.083
W*G	*	ns	ns	*	ns	**	**	ns	ns	ns

Level of significance: * p ≤ 0.05; ** p ≤ 0.01; ns: not significant. Means denoted by the same letter are not statistically different, as assessed using the LSD test at p ≤ 0.05. H and L indicate high-and low-water treatments, respectively. GY, grain yield (g/barrel); ET, evapotranspiration (L/barrel); WUE, water-use efficiency (kg/m^3^); BM, aboveground biomass (g/barrel); HI, harvest index (%); NSB, number of spikes per barrel; TGW, thousand-grain weight (g); NGS, number of grains per spike; NSS, number of spikelets per spike; NIS, number of infertile spikelets per spike.

#### 3.1.2 Evapotranspiration and water-use efficiency

The values of ET and WUE in response to the different treatments are summarized in **Table 1**. WUE was significantly affected by W and G, although the effect of G×W on WUE was not statistically significant (p > 0.05). Wheat subjected to L treatment was found to have a 11.6% higher mean WUE than that under H regime. Comparing the mean ET values obtained from H treatment, a significant ET reduction of 12.78% was recorded under L treatment. Conversely, we detected no significant differences among the wheat genotypes with respect to ET (p > 0.05), whereas a significant reduction in WUE was recorded in the XM49 genotype.

#### 3.1.3 Aboveground biomass and harvest index

The BM and HI values recorded in response to the different treatments (showed in **Table 1**) clearly indicated that HI was significantly increased from 41.6% to 44.0% (p< 0.01) under L treatment, although G and G×W appeared to have no significant effects on HI. The BM of wheat subjected to L treatment was found to be significantly lower (by 7.98%, p< 0.01) than that under H treatment. Moreover, BM was significantly influenced by both G and G×W.

### 3.2 Grain quality parameters of winter wheat

Mean values of several grain quality parameters for three wheat genotypes grown under the two irrigation regimes and their ANOVA results are presented in **Table 2**. These values indicate that whereas L treatment significantly increased GN and GI, it significantly decreased GPC. Low-water regime result in a significant increase in MPV and MRS. Contrastingly, the effects of W on the MPT were not statistically significant (p > 0.05). All quality parameters were, however, significantly affected by G, with both GI and MRS of XM26 were the highest among the three genotypes examined in this study.

**Table 2:** Grain quality parameters of different winter wheat genotypes under the different irrigation treatments and results of the analysis of variance.

	GPC	GN	GI	MPT	MPV	MRS
Water(W)	**	**	**	ns	**	*
H	15.40a	1.253b	73.48b	7.924	50.73b	-4.883b
L	15.29b	1.293a	76.21a	8.361	55.65a	-4.021a
Genotype(G)	**	**	**	**	**	**
XM26	15.73a	1.182c	92.17a	11.78a	50.44b	-1.112a
XM49	15.52b	1.392a	76.43b	7.828b	58.35a	-3.915b
ZM18	14.79c	1.244b	55.93c	4.823c	50.77b	-8.330c
W*G	ns	**	**	*	ns	ns

Level of significance: * p ≤ 0.05; ** p≤ 0.01; ns: not significant. Means denoted by the same letter are not statistically different, as assessed using the LSD test at p ≤ 0.05. H and L indicate high- and low-water treatments, respectively. GPC, grain protein concentration (%); GN, grain nitrogen content (mg/grain); GI, gluten index (%); MPT, midline peak time (min); MPV, midline peak value (% torque); MRS, right of midline peak slope (% torque/min).

### 3.3 Relationships between quality parameters, yield, yield components, and evapotranspiration

**Figure 3:**
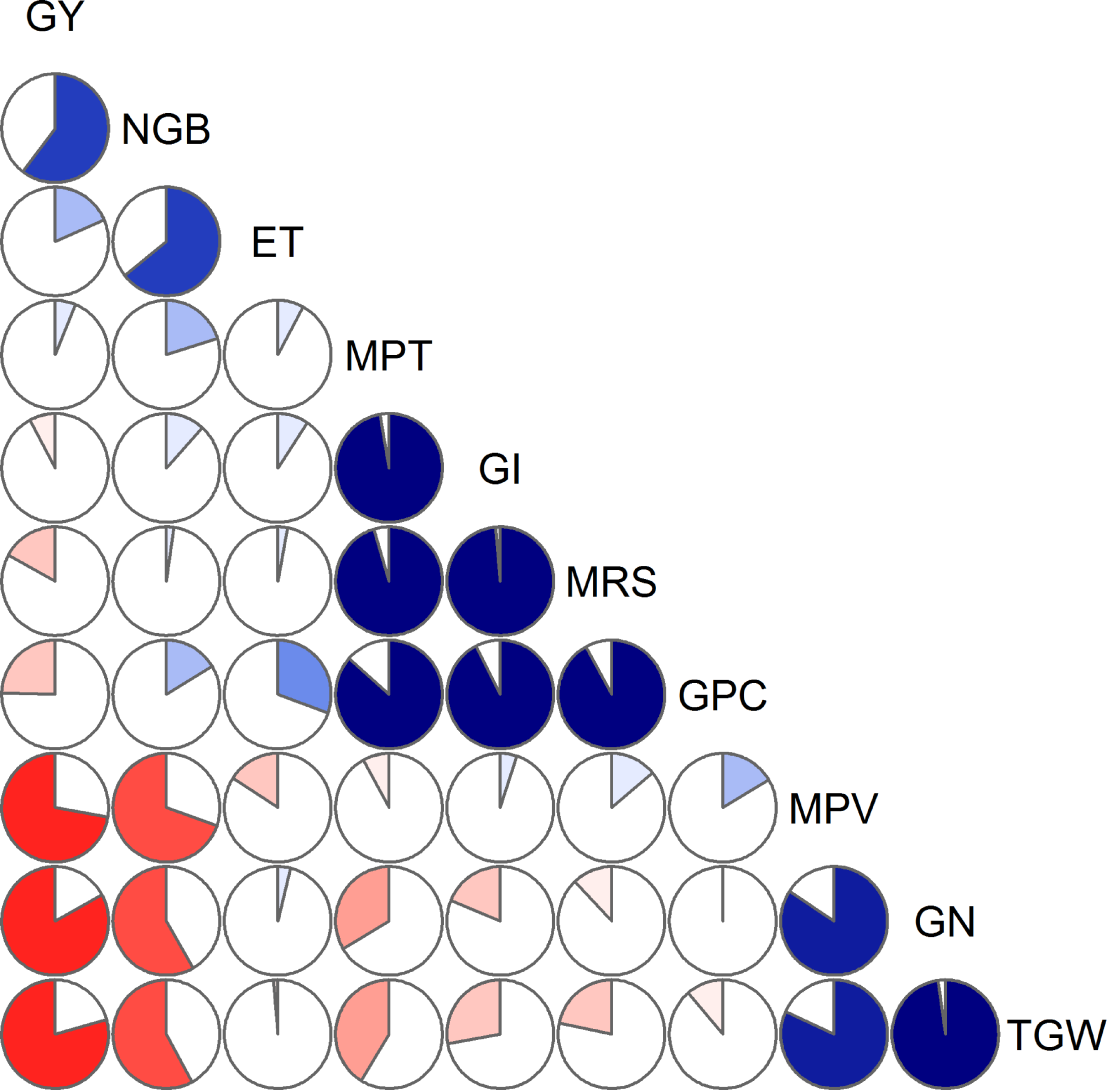
Correlation diagnosis of grain yield, evapotranspiration, and quality parameters. Blue color represents a positive correlation between the two variables that meet at that cell. Conversely, red color represents a negative correlation. The darker and more saturated the color, the greater is the magnitude of the correlation. GY, grain yield (g/barrel); NGB, number of grains per barrel; ET, evapotranspiration (L/barrel); MPT, midline peak time (minute); GI, gluten index (%); MRS, right of midline peak slope (% torque/minute); GPC, grain protein concentration (%); MPV, midline peak value (% torque); GN, grain nitrogen content (mg/grain); TGW, thousand-grain weight (g).

The observed values from all 18 samples were used to examine correlations among yield, yield components, ET, and quality traits, and the results were presented in **Table 3** and **Figure 3**. The data indicated that yield correlated negatively with TGW (r = –0.793, p< 0.01), although positively correlated with the number of grains per barrel (NGB; r = 0.603, p< 0.01). NGB was significantly positively correlated with ET (r = 0.643, p< 0.01). In addition, a significant negative correlation (r = –0.58, p< 0.05) between TGW and NGB was also recorded.

**Table 3:** Pearson’s correlation coefficients (above the diagonal) and probability values (below the diagonal) between the quality parameters, yield, yield components, and evapotranspiration of different wheat genotypes subjected to different irrigation regimes.

	GPC	GN	GI	MPT	MPV	MRS	GY	ET	TGW	NGB
GPC	1	-0.004	0.924	0.865	0.165	0.920	-0.245	0.305	-0.113	0.163
GN	0.986	1	-0.189	-0.336	0.842	-0.121	-0.830	0.039	0.977	-0.584
GI	<0.001	0.453	1	0.972	0.050	0.986	-0.078	0.093	-0.278	0.117
MPT	<0.001	0.173	<0.001	1	-0.080	0.954	0.063	0.079	-0.413	0.201
MPV	0.514	<0.001	0.843	0.754	1	0.139	-0.723	-0.158	0.819	-0.696
MRS	<0.001	0.633	<0.001	<0.001	0.583	1	-0.170	0.030	-0.217	0.023
GY	0.327	<0.001	0.757	0.805	0.001	0.501	1	0.184	-0.793	0.603
ET	0.218	0.879	0.713	0.757	0.530	0.906	0.464	1	-0.012	0.643
TGW	0.656	<0.001	0.265	0.088	<0.001	0.387	<0.001	0.961	1	-0.580
NGB	0.517	0.011	0.644	0.424	0.001	0.928	0.008	0.004	0.012	1

GPC, grain protein concentration (%); GN, grain nitrogen content (mg/grain); GI, gluten index (%); MPT, midline peak time (min); MPV, midline peak value (% torque); MRS, right of midline peak slope (% torque/minute); GY, grain yield (g/barrel); ET, evapotranspiration (L/barrel); TGW, thousand-grain weight (g); NGB, number of grains per barrel.

Correlations between MPT and GPC, between GI and GPC, and between MRS and GPC were significantly positive (p< 0.01), whereas MPT, GI, and MRS were slightly and non-significantly correlated with yield. Significant relationships were observed between GN and either MPV or TGW, with correlation coefficients of 0.842 (p< 0.001) and 0.977 (p< 0.001), respectively. Moreover, we detected a significant reduction in GN coinciding with an increase in NGB (r = –0.584, p< 0.05), whereas GN appeared to be not correlated with GPC.

### 3.4 Grain growth pattern

#### 3.4.1 Grain weight dynamic curves and parameters of grain filling

**Figure 4:**
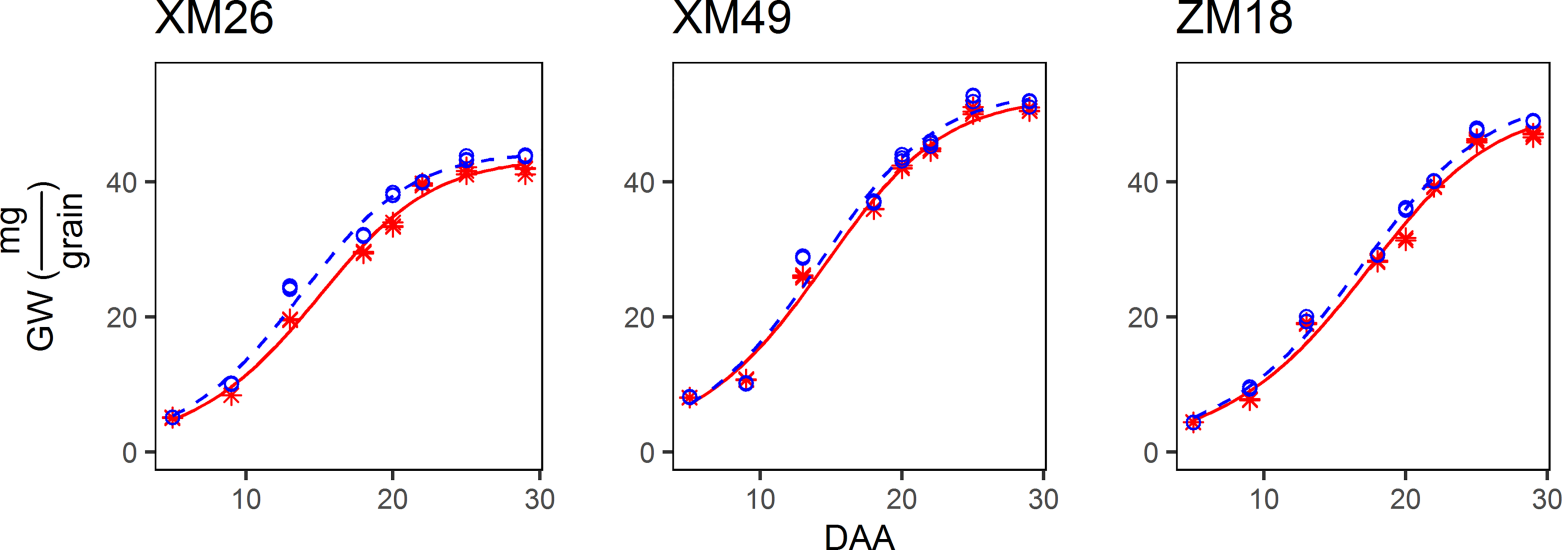
Dynamics of the grain weight (GW) of different genotypes of winter wheat under different levels of irrigation. Red lines indicate high-water treatment, blue lines indicate low-water treatment, red stars represent high-water treatment observations, and blue circles represent low-water treatment observations. DAA, days after anthesis; XM26, Xinmai-26; XM49, Xinmai-49; ZM18, Zhoumai-18.

The pattern of grain growth was suitable to be characterized by a sigmoidal curve (**Figure 4**). The correlation coefficient between the actual and fitted grain weights determined using Eq. [7] was 0.9953 (p< 0.01), which indicated that Eq. [7] provides a good estimate of the grain growth patterns observed in this study. Specifically, the differences in grain weight between the wheat subjected to L and H treatments were small during the early grain-filling period (prior to 9 DAA), and these differences gradually increased with further grain development. Moreover, a similar mode of grain growth among the three examined wheat genotypes was identified.

**Figure 5:**
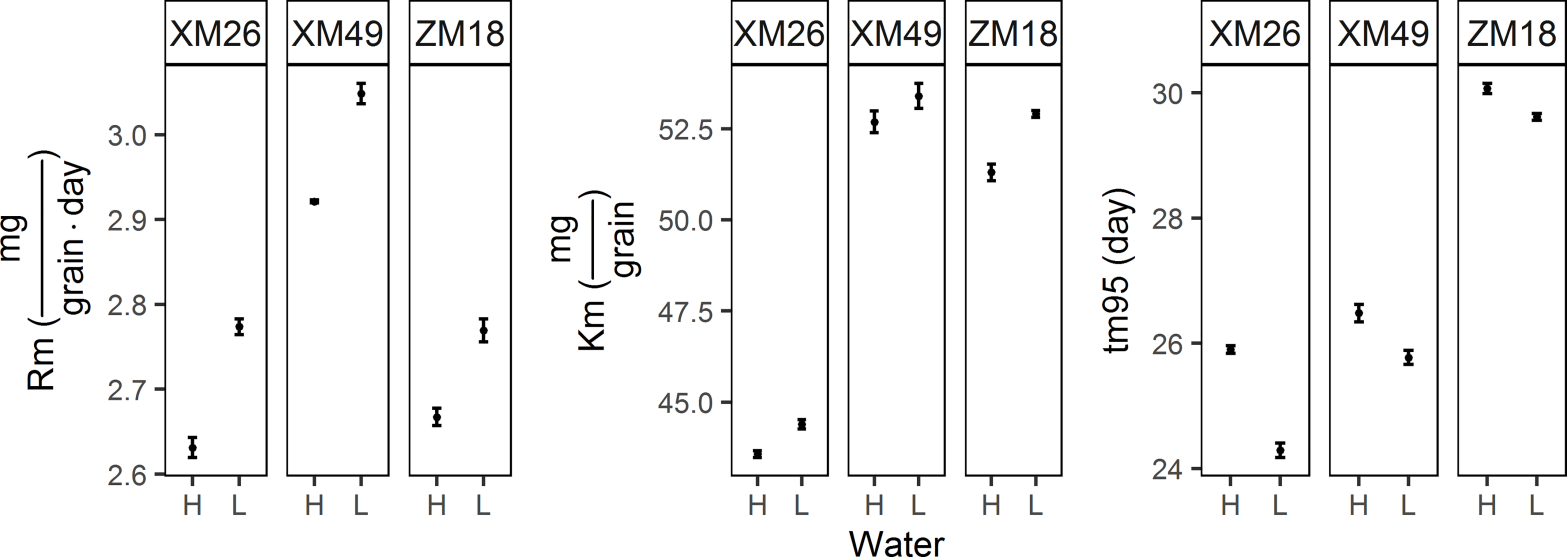
Parameters of the grain filling of different winter wheat genotypes under different irrigation levels. Error bars indicate the confidence interval of the mean. Rm, maximum rate of grain filling; Km, final grain weight; tm95, duration of grain filling. XM26, Xinmai-26; XM49, Xinmai-49; ZM18, Zhoumai-18; H, high-water treatment; L, low-water treatment.

The parameters of grain filling under the different treatments are shown in **Figure 5**. Fitted values of final grain weight (Km) for L were higher than those for H. Increases of 1.86%, 1.37%, and 3.14%, were obtained for genotypes XM26, XM49, and ZM18, respectively. When comparing the means obtained in the two irrigation regimes, we also observed an increase in maximum rate of grain filling (Rm) in response to the L treatment, with increases of 5.32%, 4.45%, and 3.75% recorded for XM26, XM49, and ZM18 respectively. We also detected reductions in the duration of grain filling (tm95) of grain filling in response to the L treatment, with reductions of 6.22%, 2.68% and 0.53% being recorded for XM26, XM49 and ZM18, respectively.

#### 3.4.2 Grain nitrogen weight dynamic curves and grain nitrogen accumulation parameters

**Figure 6:**
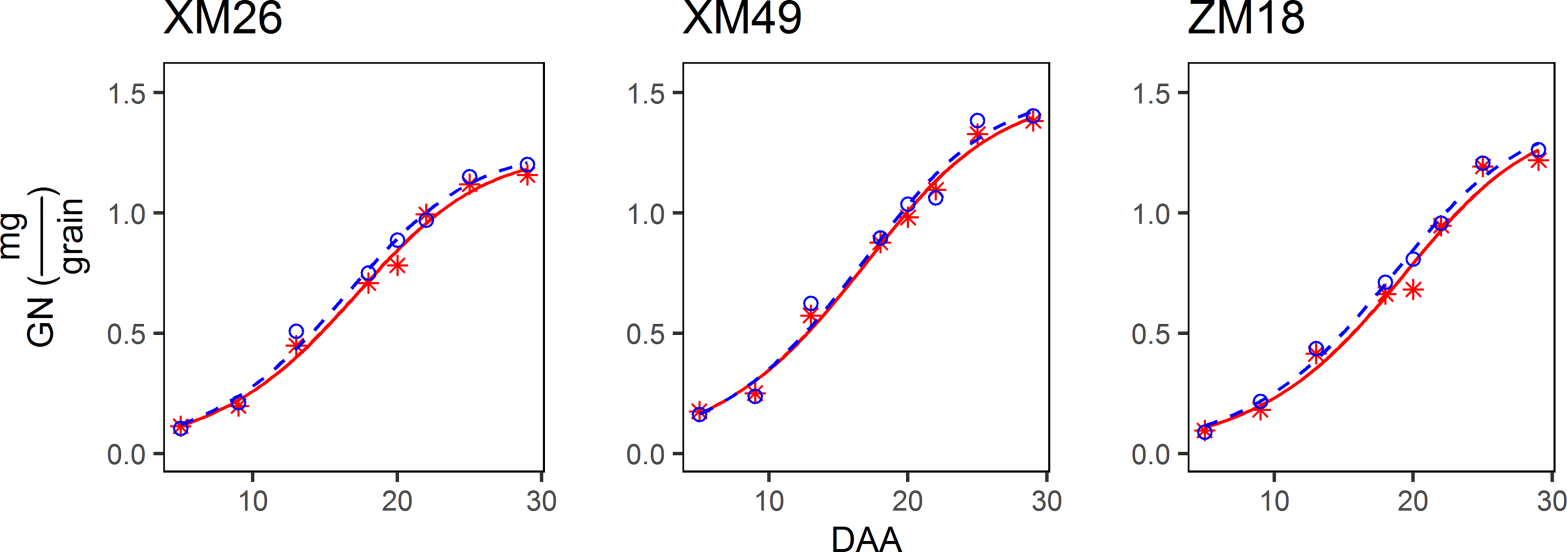
Dynamics of grain nitrogen content (GN) of different winter wheat genotypes under different irrigation levels. Red lines indicate high-water treatment, blue lines indicate low-water treatment, red stars represent high-water treatment observations, and blue circles represent low-water treatment observations. DAA, days after anthesis; XM26, Xinmai-26; XM49, Xinmai-49; ZM18, Zhoumai-18.

Patterns of grain N accumulation can be described as sigmoidal curves (**Figure 6**). In present study, the correlation coefficient between actual grain N weight and the grain N weight fitted from Eq. [7] was 0.9930 (p< 0.01), which indicated the dependability of Eq. [7] in describing the grain N weight dynamics observed in this study. Moreover, we also found that the observed pattern of grain N accumulation was similar to that of the grain growth. The differences between the GN values recorded under L and H treatments were generally small during the early grain-filling period (prior to 9 DAA). Overall, among the three wheat genotypes examined, the GN values obtained in L treatment were higher than those in H treatment.

**Figure 7:**
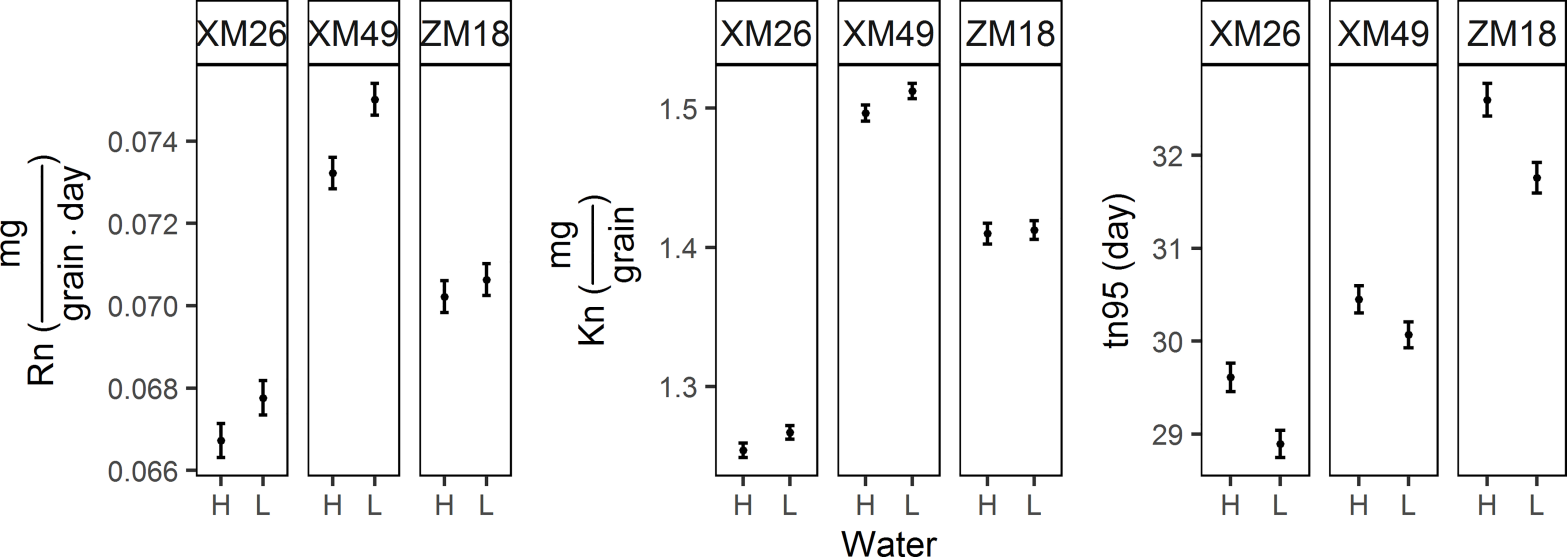
Parameters of grain nitrogen accumulation of different winter wheat genotypes subjected to different irrigation treatments. Error bars indicate the confidence interval of themean. Rn, maximum rate of grain nitrogen accumulation; Kn, final grain nitrogen weight; tn95, duration of grain nitrogen accumulation. XM26, Xinmai-26; XM49, Xinmai-49; ZM18, Zhoumai-18; H, high-water treatment; L, low-water treatment.

The grain N accumulation parameters recorded in different treatments are presented in **Figure 7**. Relative to the H treatment, the fitted values of the final GN (Kn) under L treatment increased by 1.01%, 1.05%, and 0.17% for XM26, XM49, and ZM18, respectively. Similarly, maximum rate of grain nitrogen accumulation (Rn) increased by 1.55%, 2.45% and 0.59% for the three genotypes of XM26, XM49, and ZM18, respectively. In contrast, duration of grain nitrogen accumulation (tn95) decreased by 2.42%, 1.26%, and 2.58% under L treatment for XM26, XM49, and ZM18, respectively.

### 3.5 Dynamics of sucrose and nitrogen concentration

#### 3.5.1 Dynamics of grain nitrogen concentration

**Figure 8:**
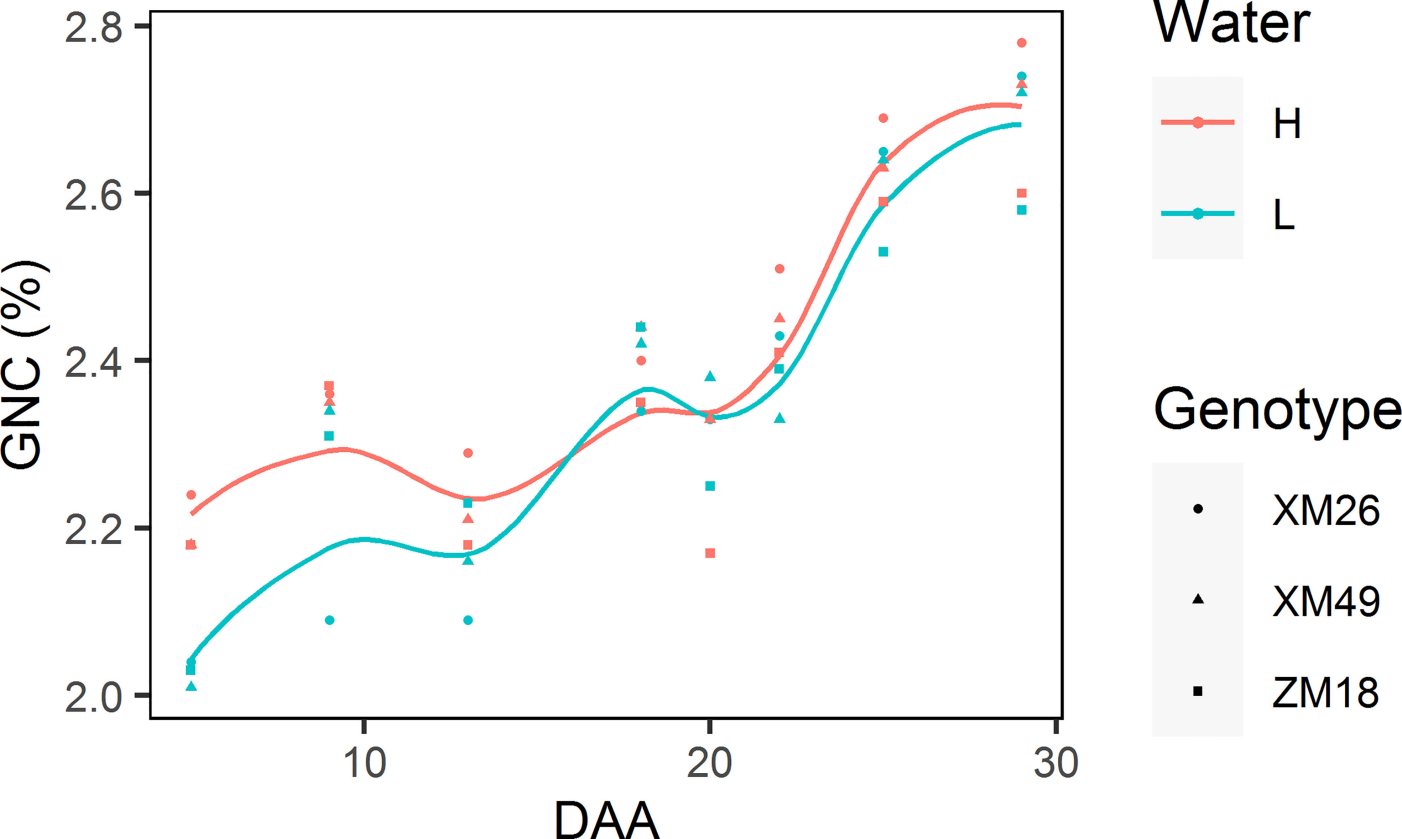
Dynamics of grain nitrogen concentration (GNC) of different winter wheat genotypes subjected to two different irrigation treatments. Lines represent values fitted using the “loess” method and the points denote observations. DAA, days after anthesis; H, high-water treatment; L, low-water treatment; XM26, Xinmai-26; XM49, Xinmai-49; ZM18, Zhoumai-18.

**Figure 8** shows the dynamics of grain N concentration (GNC). It was observed that GNC was characterized with a gradual increase and a significant fluctuation.

Moreover, with the exception of 18 DAA, the GNCs under L treatment were invariably lower than those under H treatment. During the early grain-filling period (prior to 13 DAA), we detected a great difference between the GNCs recorded in wheat under the L and H treatments. However, during the late grain-filling stage (after 18 DAA), there was a more rapid increase of GNC under L treatment, which narrowed notably the difference between the GNC values from the two different irrigation treatments consequently.

#### 3.5.2 Dynamics of leaf and rachis nitrogen concentrations

**Figure 9:**
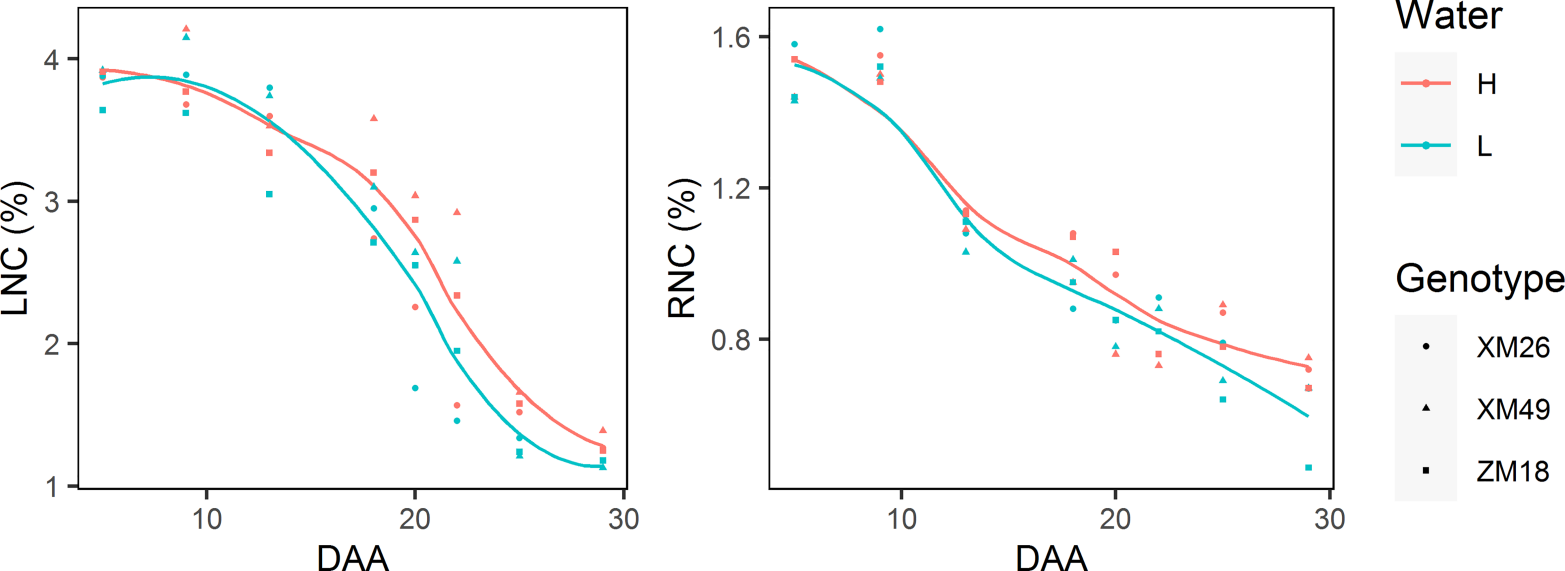
The dynamics of leaf nitrogen concentration (LNC) and rachis nitrogen concentration (RNC) of different winter wheat genotypes grown under different levels of irrigation. Lines represent values fitted using the “loess” method and the points denote observations. DAA, days after anthesis; H, high-water treatment; L, low-water treatment; XM26, Xinmai-26; XM49, Xinmai-49; ZM18, Zhoumai-18.

Both leaf and rachis nitrogen concentrations (LNC and RNC, respectively) can be characterized by gradual reductions during grain development (**Figure 9**). In most cases, the LNC and RNC of wheat subjected to L treatment were lower than those under H treatment, which became particularly apparent after 18 DAA duo to the rapid LNC and RNC reductions under L treatment.

#### 3.5.3 Dynamics of leaf and rachis sucrose concentrations

**Figure 10:**
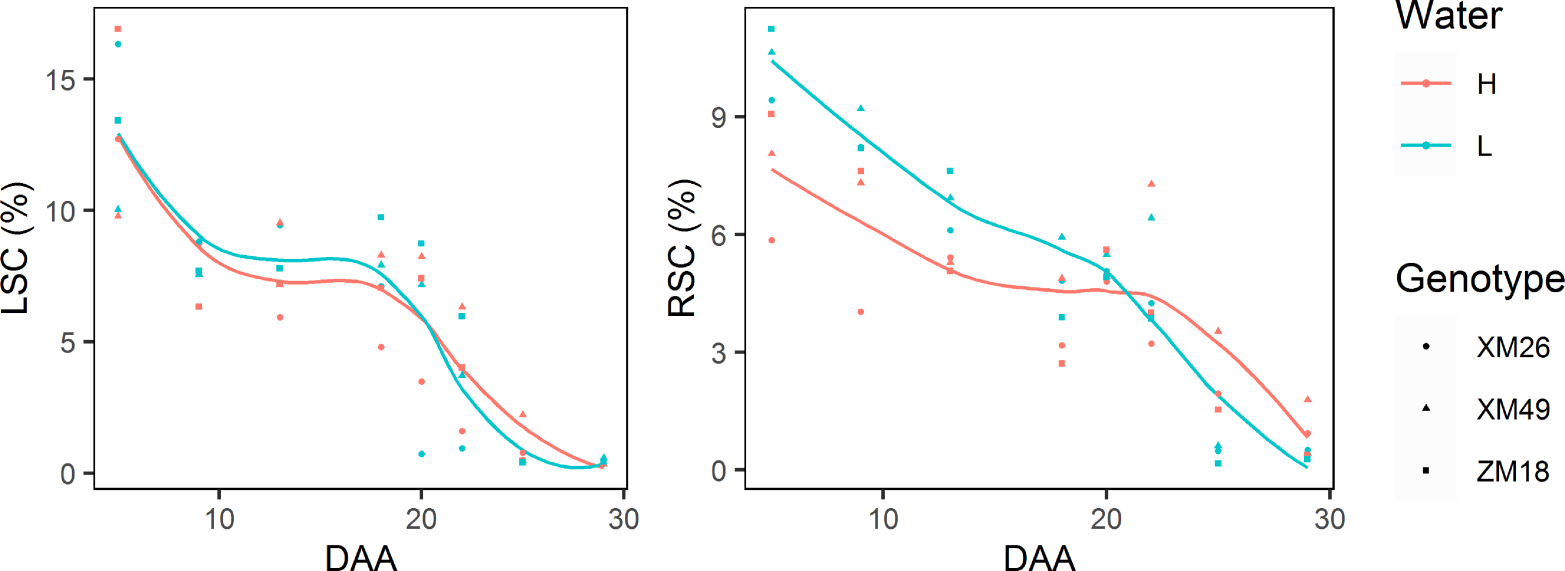
The dynamics of leaf sucrose concentration (LSC) and rachis sucrose concentration (RSC) in different winter wheat genotypes under different levels of irrigation. Lines represent values fitted using the “loess” method and points denote observations. DAA, days after anthesis; H, high-water treatment; L, low-water treatment; XM26, Xinmai-26; XM49, Xinmai-49; ZM18, Zhoumai-18.

As shown in **Figure 10**, the leaf and rachis sucrose concentrations (LSC and RSC, respectively) of the three wheat genotypes reduced gradually during grain development stage. During the early stages of grain filling (prior to 18 DAA), the LSC under L treatment was higher than that under H treatment. After 18 DAA, however, a rapid decrease of LSC was record, with the rate of reduction being more pronounced under L treatment. Consequently, the LSC value measured at 20 DAA under L treatment was lower than that under H treatment. Under H treatment, RSC declined rapidly at 23 DAA, which was later than the decline observed in response to the L treatment (20 DAA). Meanwhile, after 23 DAA the RSC of wheat subjected to L treatment was lower than that of H treatment plants.

## 4 Discussion

### 4.1 Effects of drought stress on the yield and the mechanisms whereby water management alters wheat yield

Studies that have examined the effects of drought stress on wheat yield have tended to obtain inconsistent results, and Farooq et al. (2017) suspect that these discrepancies could reflect differences in the times at which stress occurred and/or differences in the durations and intensities of the stress experienced. For example, Mu et al. (2021a) found that short-term drought after the jointing period reduced the yield of wheat by 2.03% - 64.39% compared with that of wheat receiving an adequate supply of water. Similarly, Santis et al. (2021) found that drought stress could lead to poor grain sets and yields, and Li et al. (2021) recorded low yields in plants subjected to low-level irrigation. However, it has previously been reported that a slight or moderate water deficit prior to the stem elongation stage and slight water deficit during the grain-filling stage did not significantly reduce grain yield, which lead to a significant water conservation (Meng et al., 2016). Slight and moderate water deficits can be defined as 60% to 65% and 50% to 55% of FC, respectively. Results obtained in previous studies by Yan et al. (2019; 2020) revealed that a mild water deficit can cause a slight, although non-significant, reduction (2.58%) in the yield of winter wheat, which is consistent with our findings in the present study. We established that low-water treatment non-significantly reduced the yield of wheat by less than 3%.

The number of grains per m^2^ has been identified as the main determinant of grain yield in wheat (Beral et al., 2020). In the present study, we found that yield reductions were associated with declines in the NGB. Our findings tend to indicate that drought stress experienced during the jointing stage reduces the survival rates of tillers and harvestable spikes. Although drought stress during the booting stage inhibited spikelet development and reduced the number of spikelets, we established that the effects on the NIS were non-significant. These findings are consistent with those presented by Tatar et al. (2020), who observed drought-induced reductions in spike and spikelet numbers during the stem elongation stage. The lower NGS in wheat exposed to the low-water treatment can be assumed to reflect insufficient spikelet development. However, an increase in TGW under low-water conditions was found to have a compensatory effect on grain yield, thereby reducing yield loss.

This increase in TGW in response to low-water treatment provides an evidence of compensatory effects among the yield components and, more importantly, an increase in dry matter remobilization efficiency. In this regard, the findings of some studies (Beral et al., 2020; Sanchez-Bragado et al., 2020) have indicated that the TGW is negatively correlated with grain number. Wu et al. (2020) also found that irrigation at the jointing stage increased spike number, kernels per spike, and grain yield, but reduced grain weight. Consistently, we detected a significant negative relationship between TGW and the NGB (r = –0.58, p< 0.05) in the present study. A slight water deficit applied at the jointing stage can inhibit the growth of ineffective tillers and improve the harvest index. It has been widely reported that dry matter remobilization efficiency and the harvest index can been enhanced by drought stress (Tatar et al., 2020; Moradi et al., 2022). One benefit of soil drying at the later grain-filling stage is enhancing plant senescence, and thereby promoting a more rapid and efficient remobilization of pre-stored carbon from vegetative tissues to developing grains (Yang et al., 2000). In the present study, we detected a significant increase of harvest index from 41.6% to 44.0% in response to low-water treatment, although there was a 7.98% reduction in BM.

The variation in TGW was confirmed by the fitted value of the re-parameterized Richards growth model. Richards and Logistic sigmoidal curves are commonly utilized to model organism and kernel growth (Raobert et al., 1999; Zahedi and Jenner, 2003; Yang et al., 2004; Tjørve and Tjørve, 2010; C. E. Timothy Paine et al., 2012; Wu et al., 2018). The Richards equation is a generalized form of the logistic function that has considerable flexibility when analyzing responses under stress conditions (Richards, 1959; Tjørve and Tjørve, 2010). With the inclusion of the tm95 and Rm of grain filling, the re-parameterized Richards growth model is more convenient when used to fit the grain filling process. In previous studies, it has been revealed that “controlled soil drying” at the later stage of grain filling can significantly increase the rate of grain filling and grain weight (Yang and Zhang, 2005). Although severe post-anthesis water deficit can reduce the duration and rate of dry mass accumulation and grain weight (Triboï et al., 2003; Ercoli et al., 2008; Yang et al., 2016), grain filling can be significantly promoted by a moderate post-anthesis water deficit (Zhang et al., 2012; Yang et al., 2016). In the present study, we found that Rm increased significantly in response to low-water treatment in all three wheat genotypes, which may be a good evidence in support of the opinion that Rm is responsible for increases in grain weight.

The rate of grain filling appears to reflect the rate of biochemical reactions involved in the synthesis of starch deposited in the grains (Jenner et al., 1991). In this regard, our examination of sucrose concentrations in wheat subjected to high- and low-water treatments tended to indicate that lower soil moisture promotes the accumulation of starch in grains. The concentration of sucrose in leaves reflects the progress of photosynthesis and the rachis has been identified as providing a temporary store of carbohydrates assimilated *via* leaf photosynthesis. On the commencement of grain filling, the leaves begin to senesce and sucrose stored in leaves and rachis is transferred to the grain. Consequently, gradual reductions in the concentrations of leaf and rachis sucrose are detected concomitant with the progression of grain filling. In the present study, we found that during the early stage of grain filling, the concentrations of sucrose in the leaves of wheat subjected to the low-water treatment were higher than those in the leaves of wheat receiving a higher level of irrigation, although the rate of decline was higher during the late stage of grain filling. During leaf senescence, carbohydrates deposited in the leaves are transferred to the grain, and the more rapid the reduction in sucrose concentration, the faster is the transfer of carbohydrate. Compared to wheat plants under high-water treatment, the rapid decline in rachis sucrose concentration was detected at an earlier stage of grain development in wheat subjected to low-water treatment. It has been previously reported that drought stress increases the remobilization of carbon reserves from vegetative tissues to grains (Farooq et al., 2017), but the duration of grain filling decreases with an increase in water deficit. The grain-filling rate has been found to increase in response to a mild water deficit (Yan et al., 2019). The aforementioned observations thus indicate that a higher rate of photosynthesis during the early stage of grain filling and a higher rate of carbohydrate transfer from vegetative organs to grains during the later stage are the main factors associated with an increase in grain filling rate and grain weight in wheat subjected to low-level irrigation.

### 4.2 Effects of drought stress on the end-use functional properties of wheat

During the growth period, drought stress affects the concentration of grain proteins, and variations in the concentration of these proteins is typically dependent on the intensity of the stress experienced (Ozturk et al., 2021). However, the relationship between stress intensity and protein concentration is non-linear, with drought stress experienced during the growth period generally promoting an increase in GPC (Barutçular et al., 2016; Rakszegi et al., 2019; Tatar et al., 2020; Kamara et al., 2022). Moreover, there is a negative association between GPC and total irrigation amount (Moradi et al., 2022). Wu et al. (2020) also found that the concentration of grain protein was significantly increased by a water deficit during the grain-filling stage. However, the effects of drought at the grain-filling stage were found to differ according to stress intensity, with a slight water deficit significantly reducing GPC, whereas severe water deficit has been found to increase the concentration of grain proteins during the grain-filling stage (Meng et al., 2016). Furthermore, the findings of some studies have indicated that the concentration of grain proteins initially increases and subsequently declines in response to an increase in drought stress degree (Silva et al., 2020; Yan et al., 2020). In the present study, we detected reductions in protein concentrations in the grains of wheat subjected to the low-water treatment, which is consistent with the findings of Meng et al. (2016), but contrasts with observations of Silva et al. (2020); Tatar et al. (2020), and Yan et al. (2020). We suspect that these discrepant findings could reflect differences in the respective intensities of the applied stresses.

The GI, an indicator of gluten strength, is predominantly determined by genotype (Vida et al., 2014; Sekularac et al., 2018; Kun et al., 2020), although can also be significantly influenced by irrigation, precipitation, and soil moisture content (Gil et al., 2011; Oikonomou et al., 2015). Generally, drought stress is associated with an increase in the GI. For example, Ames et al. (2003) have found that the GI appeared to be slightly but significantly higher under dry conditions than under irrigated conditions for all genotypes used in their study. Moreover, Flagella et al. (2010) established that drought stress occurred in the late grain-filling stage increases gluten strength, and Li et al. (2013) also found that gluten strength is higher under drought stress conditions. Consistently, the findings of some studies have revealed that gluten strength is higher in severe drought environments compared with full irrigation (Hernandez-Espinosa et al., 2018). In the present study, we recorded an increase in the GI of wheat subjected to the low-water treatment, which contrasts with the findings of Yang et al. (2018), who revealed that the GI was not significantly affected by drought stress, although the highest GI value was recorded in plants subjected to mild drought stress. This discrepancy could be attributed to differences in the intensities of the drought stress applied and/or the genotypes of wheat examined in previous studies. Whereas a negative correlation between GPC and the GI has been detected in some previous studies (Ames et al., 2003; Yang et al., 2018), Kun et al. (2020) observed a slightly positive, albeit statistically non-significant, relationship between GPC and the GI. In the present study, however, we found GI values to be significantly positively correlated with GPCs. We suspect that the differences in the finding of these studies could be associated with differences in the genotypes of wheat assessed. The three genotypes examined in the present study were selected according to dough strength, and the sample space was insufficiently large. In contrast, previous studies have typically examined a more extensive range of genotypes, for which protein concentrations varied considerably.

Dough elasticity exhibited as a rubber can be significantly increased by light or moderate drought stress. In previous studies, the MPT (Li et al., 2013; Hernandez-Espinosa et al., 2018; Yang et al., 2018), MPV (Labuschagne and Moloi, 2015; Yang et al., 2018) and midline peak integral (Labuschagne and Moloi, 2015; Hernandez-Espinosa et al., 2018) have been shown to increase in response drought stress. MRS (negative value), which is negatively correlated with midline peak integral, is used as an indicator of the degree of dough softening. Our findings with respect to dough elasticity are generally in accordance with those reported previously, although we detected a slight, albeit non-significant, increase in MPT, which can be attributed to differences in the degree of applied stress. In general, strong gluten dough is associated with a high MPT and low absolute values of MRS (Mao et al., 2013), and in the present study, we found that the MPT and MRS (negative values) also exhibited significant positive relationships with GPC.

### 4.3 Possible causes for the higher grain quality under low-water treatment

GN is a reliable indicator of dough elasticity and can be used to determine the proportional composition of grain proteins (Martre et al., 2003; Ferrise et al., 2015; Mefleh et al., 2020; Snowdon et al., 2021). Consequently, changing GN will inevitably modify the proportional composition of grain proteins, which is determined by the “allometric scaling” relationships among the protein compositions. The ratio of the grain protein composition (i.e., gliadin to glutenin) determines the balance between dough viscosity and elasticity. In the current study, we found that MPV was positively correlated with GN (r = 0.842, p< 0.001), although showed a non-significant correlation with GPC (r = 0.165, p = 0.514). Moreover, we detected no evident correlation between GN and GPC levels, which is conceivably attributable to a synchronous increase in GN and TGW, and the change in the ratio of these two parameters became smaller. When comparing only the means of the water treatments, we established that the low-water treatment promoted simultaneous increases in GN, GI, MPV, and MRS (negative values). Accordingly, it can be speculated that drought stress enhances gluten strength and dough elasticity *via* its effect on increasing GN. In this context, studies on the molecular weight distribution of grain proteins are required to confirm the relationship between GN and protein polymerization. Some studies have shown that grain N accumulation is source-regulated (Martre et al., 2003; Zhao et al., 2021), and GN has been observed to increase significantly in response to a reduction in grain number per m^2^. The results obtained in the present study revealed that GN is negatively correlated with NGB, which is consistent with the findings of previous studies.

An increase in the amount of GN is assumed to be associated with an increase in grain Rn promoted by low-water treatment. In turn, an increase in Rn indicates that the nitrogen supplied to the grains is sufficient. The findings of previous studies have revealed that mild water deficits promote N availability and uptake (Széles et al., 2012; Yan et al., 2020), whereas irrigation at the anthesis and grain-filling stages has been found to cause reductions in the N harvest index (Moradi et al., 2022). GN is primarily determined by the N stored in the leaves at anthesis (Zheng et al., 2020), and during the grain-filling stage, proteins deposited in the leaves are broken down into free amino acids that are remobilized to the grain *via* rachis (Zhao et al., 2015; Hecke et al., 2020). In response to water deficit, the leaves of wheat plants at the late grain-filling stage undergo premature senescence, with concomitant rapid reductions in the concentration of leaf and rachis N. The process of leaf senescence, reductions in the sucrose and N concentration in leaves, and increases in GNC occur synchronously, and Gomez-Sanchez et al. (2019) have established that a delay in leaf senescence in barley resulted in lower amounts of seed N due to a reduction in leaf N remobilization. In the present study, we found that the low-water treatment triggered an earlier rapid transfer of N deposited in vegetative organs. At the late grain-filling stage, we observed that the decline in the N content of leaves in response to low-water treatment occurred at an earlier time point than the reduction in sucrose content. Given that GNC (**Figure 7**) is affected by the amount of grain starch, it tends to be characterized by considerable fluctuations, and accordingly, would be unsuitable as a predictor of grain quality.

### 4.4 Effects of drought stress on grain yield and quality are also genotype-dependent

Genetic variations in the responsiveness of wheat genotypes to drought stress have previously been reported (Farooq et al., 2014; Yang et al., 2016; Moradi et al., 2022), and it has been established that drought-resistant genotypes can maintain appropriate functionality in a severely dehydrated state by producing a well-developed root system, initiating osmotic adjustment, and remobilizing water-soluble stem carbohydrates. In this regard, it has been proven that the effects of drought stress on TGW vary according to genotype (Yang et al., 2016; Silva et al., 2020; Rezzouk et al., 2022), and Silva et al. (2020) have similarly established that the effects of water deficit on protein content in grains are genotype dependent. In the present study, we found that the wheat genotype ZM18 was characterized by the smallest reduction in duration (tm95), and the most pronounced increase in grain weight (Km), which thereby indicates that ZM18 has a higher adaptability to drought stress than the other two assessed wheat genotypes. However, we also established that ZM18 was characterized by the smallest increase in Kn (GN fitted value) and Rn, thus indicating that it would be difficult to improve grain N accumulation in this genotype. Strong gluten genotypes are characterized large increases in Kn and Rn in response to water deficit, which indicates that grain N accumulation could be readily enhanced in these genotypes. These findings thus confirmed that the effects of drought stress on grain yield and quality are genotype dependent, and accordingly, further studies are required that seek to identify the effects of drought-by-genotype interactions on grain yield and quality in wheat using a more extensive selection of genotypes.

### 4.5 Relationships between grain yield, quality, and WUE

In this study, we detected no significant relationship between wheat yield and ET. However, we observed a significant enhancement of WUE in response to low-water treatment, which is consistent with the findings of Mu et al. (2021b). The findings of some previous studies have revealed that yield and grain weight are negatively correlated with GPCs (Souza et al., 2004; Nehe et al., 2020; Ali and Akmal, 2022). Although in the present study, we also detected negative correlations between yield and grain weight and GPC, the associations were comparatively slight and non-significant, which is consistent with the findings of Li et al. (2013). In contrast, however, Yan et al. (2020) have previously reported a positive relationship between grain yield and GNC. These discrepancies in the findings of different studies are assumed to be attributable to differences in the intensities of the applied stress and assessed genotypes. Furthermore, given that the correlation coefficients obtained for the associations between wheat yield and the parameters GI, MPT, and MRS were not statistically significant, it is conceivable that the negative relationship between yield and grain quality could be altered, and that by optimizing water management and variety selection, we can achieve high yield, high quality, and water-saving wheat production targets.

Our findings in this study indicate that the end-use functional properties of wheat are influenced by water management, and that RDI may be used to enhance wheat quality and WUE without incurring yield loss. A moderate water deficit (60% to 65% FC) applied prior to the jointing stage and at the late grain-filling stage, combined with a slight water deficit (65% to 70% FC) from jointing to booting was found to increase grain quality and WUE. The non-significant reduction of less than 3% in the yield of wheat subjected to RDI can be attributed to a higher photosynthetic rate during the early stage of grain filling and a higher rate of carbohydrate transfer from vegetative organs to grains during the later stage under low-water treatment. We speculate that RDI might contribute to enhancing grain quality *via* an increase in GN, whereby RDI triggers an earlier rapid transfer of N deposited in vegetative organs, resulting in a synchronous reduction in the N concentration in leaves and an increase in GNC. Furthermore, our observations of a positive correlation between MPV and GN, indicate that by increasing GN, it would be possible to enhance dough elasticity. Given that the effects of RDI on grain quality were genotype dependent, further studies on the interactive effects of genotypes and water management are required.

## Data availability statement

The raw data supporting the conclusions of this article will be made available by the authors, without undue reservation.

## Author contributions

DA and SK conceived and designed the experiments. SK conducted field experiments. SK and XL (physiological parameters) conducted laboratory analyses and wrote the manuscript. WM and LH analyzed the data and revised manuscript. All authors contributed to the article and approved the submitted version.

## Funding

This study was financed by the Ministry of education of the PRC (grant number ZH2021040101), the Ministry of Science and Technology of PRC (grant number 2018YFD0300703) and China Agriculture Research System project (grant number CARS-3).

## Conflict of interest

The authors declare that the research was conducted in the absence of any commercial or financial relationships that could be construed as a potential conflict of interest.

## Publisher’s note

All claims expressed in this article are solely those of the authors and do not necessarily represent those of their affiliated organizations, or those of the publisher, the editors and the reviewers. Any product that may be evaluated in this article, or claim that may be made by its manufacturer, is not guaranteed or endorsed by the publisher.
